# Preparation of Phase Change Microcapsules with the Enhanced Photothermal Performance

**DOI:** 10.3390/polym11091507

**Published:** 2019-09-16

**Authors:** Sara Tahan Latibari, Jacco Eversdijk, Ruud Cuypers, Vassiliki Drosou, Mina Shahi

**Affiliations:** 1 Faculty of Engineering Technology, Department of Thermal and Fluid engineering (TFE), University of Twente, 7500 AE Enschede, The Netherlands; 2 TNO, 5656 AE Eindhoven, The Netherlands; jacco.eversdijk@tno.nl; 3 TNO, 2628 CA Delft, The Netherlands; ruud.cuypers@tno.nl; 4 Solar Thermal Systems Department, Center for Renewable Energy Sources, Pikermi, 19009 Athens, Greece; drosou@cres.gr

**Keywords:** phase change material, micro-encapsulation, polyurea, graphite nanoplatelet, thermal storage, photothermal

## Abstract

The performance of solar-thermal conversion systems can be improved by incorporation of encapsulated phase change materials. In this study, for the first time, Crodatherm^TM^ 60 as a phase change material (PCM) was successfully encapsulated within polyurea as the shell supporting material. While preparing the slurry samples, graphite nanoplatelet (GNP) sheets were also incorporated to enhance the thermal and photothermal properties of the prepared materials. The morphology and chemical properties of these capsules were characterized by scanning electron microscopy (SEM) and Fourier transform infrared (FTIR) spectrum, respectively. The results show the spherical-like and core-shell structure of capsules with an average diameter size of 3.34 μm. No chemical interaction was observed between the core and the supporting materials. The thermal characteristics of the microencapsulated PCMs (MEPCMs), analyzed by differential scanning calorimetry (DSC) and thermogravimetric analysis (TGA), indicate that the prepared samples with 0.1 weight percentage of GNP possess the latent heat of 95.5 J/g at the phase transition temperature of about 64 °C. Analyzing the rheological properties of the prepared slurry with 16 wt % of MEPCMs proves that the prepared material meet the requirements given by the heat transfer applications. The thermal storage capacity, good thermal stability, and improved photothermal performance of the prepared material make it a potential candidate for using in direct absorption solar thermal applications.

## 1. Introduction

Utilization of solar energy is a promising sustainable solution to the energy crisis and environmental protection of its free availability and least environmental affect [1]. Despite its attenuation, the total amount of available solar energy is still massive. However it is necessary to capture, convert, and store the solar energy efficiently due to the low-density and intermittency of this type of energy source [2]. Solar thermal collectors, which are the essential component of any solar thermal system, absorb the solar radiation and transform it into useful thermal energy [3]. A typical solar thermal system consists of a transparent cover, an absorber, insulation in the back, and slide sides and the frame. To simplify the construction of typical solar thermal collectors and improve the optical efficiency as well as overcome the heating losses due to the cover, a so-called direct absorption solar collector (DASC) has been introduced [4]. DASC belongs to the wind and irradiance sensitive collectors (WISC) category of solar thermal collectors. In a DASC, solar energy is directly absorbed and transferred by the heat transfer fluid. However, the efficiency of DASCs is limited by the photo thermal characteristics of the working fluid, which is very poor for typical fluids such as water and oils [5]. In addition, most of the heat transfer fluids have some limitations like the light-induced degradation and poor thermal stability as well as low thermal conductivity. Adding small amounts of solid particles with high thermal conductivity is an effective method to enhance the thermal conductivity of fluids [6]. Therefore, the nanofluids containing some beneficial energy-efficient photo-thermal materials like polymeric materials (polypyrrole, polyaniline), plasmonic nanoparticles (gold, silver, platinum), metal and metal oxide nanoparticles (copper, alumina), and carbon-based materials (graphite nanoparticles, carbon nanotubes, carbon nanohorns, carbon black, graphene) seem to be promising additives in DASC systems [7]. Carbon-based materials like crystalline (diamond), graphite sheets (graphite nanoplatelet), carbon nanotubes, and carbon nanoparticles are ideal additives for heat transfer fluids due to their high thermal conductivity as well as their black color [8,9]. Otanicar et al. [10] fabricated different types of nanofluids for the DASC system with water as a base fluid, along with graphite (30 nm diameter), carbon nanotube (6–20 nm diameter), and silver (20 and 40 nm diameters) as the additives. By applying the above mentioned nanofluids in solar thermal collectors they achieved an efficiency enhancement of up to 5%. Ladjevardi et al. [11] utilized graphite-water nanofluid with a volume fraction of around 0.000025% in a solar thermal collector. They realized that more than 50% of the incident irradiation energy can be absorbed, while the water-based solar collector only absorbed around 27% of the incident irradiation energy under the same conditions.

To increase the collector efficiency, enhancement in the desired output temperature of the system is required. However, the specific heat of the nanofluid is low in comparison with the base fluid [12,13]. This means that a high effective receiver efficiency can be achieved at high levels of radiation. Moreover, the limitation in the time mismatches between the availability of solar energy and the demand negatively affecting the efficiencies of currently available solar collectors [14]. Therefore, an interesting idea to overcome the above-mentioned limitations is to add PCMs to the heat transfer fluid and hence exploit the latent heat in combination with the sensible heat of the carrier [15].

Recently, PCMs have been widely used in heat transfer fluids due to their capability of storing and releasing a great amount of thermal energy during the phase transition [16]. This kind of heat transfer fluid has a greater evident specific heat in the phase transition temperature. Due to the influence of phase change particles on fluid flow and heat transfer, the heat transfer capability of the fluid can be significantly increased [17,18]. Despite the excellent advantages in using organic PCMs, they still suffer from some difficulties including inherent low thermal conductivities. This disadvantage increases the charging and discharging time significantly and causes a low solar harvesting efficiency. Next to this, the seepage of liquid-state of organic PCMs can result in the contamination of equipment as well as the loss of PCM [19]. Micro/nanoencapsulation of PCMs (ME/NEPCMs) within different types of shell materials is proposed to overcome these problems [20]. ME/NEPCMs can significantly increase the heat transfer coefficient and enhance the surface-to-volume ratio of phase change materials. The shell of microcapsules reduces PCM reactivity with the outside environment and controls the volume changes as the phase change occurs. Polymeric shell materials are the most widely used shell materials due to their outstanding properties [21]. Polymeric microcapsules of PCMs have been facilely realized through an emulsion or suspension polymerization route, and the resulting polymeric shells can not only provide an effective protection as well as a stable form/shape for the encapsulated PCMs but also increase the surface-to-volume ratio to improve heat transfer for them [22].

In recent years, several research studies have been performed to examine the effects of applying PCMs on the performance of DASC systems [23]. Beside the heat storage capacity, the efficiency will be significantly improved if PCMs also possess the capability of absorbing solar irradiation and transferring it into thermal energy [24]. Wang et al. [25] reported that the achieved working temperature of illuminated dispersion of graphite nanoparticle in paraffin/water emulsion ranges from room temperature to 80 °C. They obtained a receiver efficiency of 86.8% and energy density of two times as high as that of water by utilizing the PCM emulsion containing 0.1 wt % graphite. Yuan et al. [26] fabricated paraffin@silica/graphene oxide (GO) microcapsule water-based slurry that exhibits a remarkable increase in specific heat compared with water. In another study, Xu et al. [27] confirmed the enhanced light absorbing properties, thermal conductivity, and photo-thermal conversion performance of paraffin@Cu-Cu_2_O microcapsule slurry compared to the paraffin emulsion.

In this paper, a novel microencapsulated PCM slurry containing graphite nanoplatelet was prepared by using the interfacial polymerization method. Due to the common issues in preparing interfacial encapsulation, such as formaldehyde residue in microcapsules shells, polyurea as an optimally used polymer were selected to fabricate MEPCMs. The thermophysical and photothermal properties of the prepared MEPCMs were investigated for utilization in DASC.

## 2. Materials and Methods 

### 2.1. Materials

CrodaTherm™ 60 (Cr60) with melting temperature of 59.8 °C as the thermal storage segment was kindly provided by Croda B.V. Gouda, Netherlands. The interfacial reaction was done by using isopherone-di-isocyanate (IPDI, 99 wt %) and diethylenetriamine (DETA, 99 wt %) which were obtained subsequently by BAYER, Maastricht, Nethelands and Sigma Aldrich. Polyvinyl alcohol 18-88 (PVA) as the surfactant was purchased from Sigma Aldrich, Zwijndrecht, Netherlands. Graphite nanoplatelet aggregates (GNP-500 m^2^/g), as the solar-thermal part, was supplied by Alfa Aesar, Kandel, Germany. All the chemical reagents were of chemical pure grade and were used as received without further purification.

### 2.2. Fabrication of Cr60@Polyurea Microcapsules

The PCM was encapsulated by following an interfacial reaction using isopherone-di-isocyanate (IPDI) and diethylenetriamine (DETA). In a beaker, 75 g of molten Cr60 wax and 25 g IPDI were mixed and placed in a circulation oven at 80 °C. Then, 500 g of 1 wt % PVA 18-88 solution in demi water was prepared and kept at 80 °C. After 1 h the Cr60-IPDI mixture was poured into 1 wt % PVA solution. Subsequently the beaker was transferred to an ultrasonication probe (Analog Sonifier S450A Cell Disruptors, Branson Ultrasonic, Carouge, Switzerland) and sonicated for 3 min while stirring with a magnetic stirrer. A milky white emulsion was obtained. This emulsion was rapidly transferred to a glass double walled reactor at 80 °C equipped with a dissolver stirring device. While stirring the emulsion, 25 g DETA, dissolved in 125 g demineralized water, was added, dropwise, over 5 min.

The emulsion was subsequently stirred for another half an hour at 80 °C, cooled down to 20 °C and stirred overnight on a rolling stirrer. Finally, a milky white dispersion was obtained which was colloidal stable and did not sediment.

### 2.3. Fabrication of Cr60@Polyurea-GNP Microcapsules

**Figure 1:**
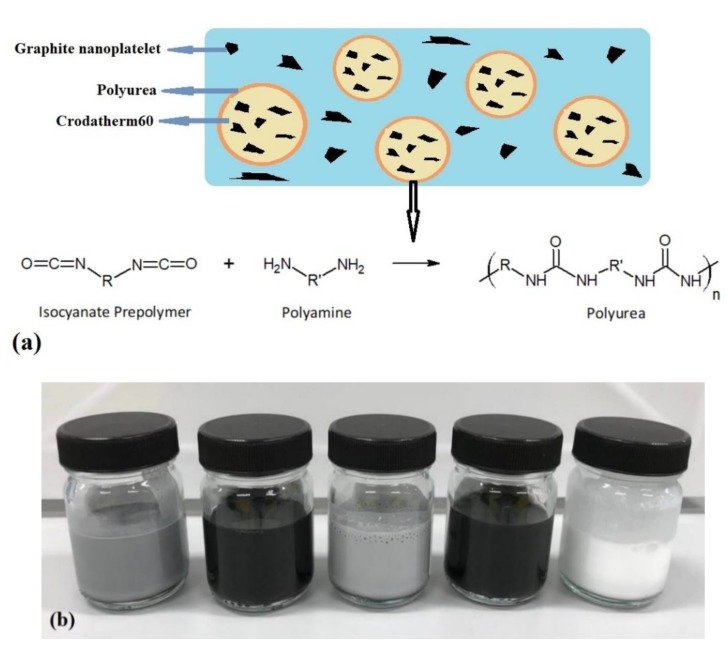
(**a**) Scheme of chemical interfacial reaction and structure of the prepared capsules; (**b**) from left to right Crodatherm60@polyurea-0.1%GNP, 0.1%GNP, Crodatherm60@polyurea-0.04%GNP, 0.04%GNP, Crodatherm60@polyurea.

For the emulsions containing GNP in the Cr60 wax capsules, GNP was mixed with Cr60 initially following the same procedure as preparation of Cr60 capsules. Moreover, emulsions are stirred after sonification for 3 h in a double walled reactor since they tend to sediment over time. It was noticed that the reaction time of half an hour at 80 °C is not sufficient to fully complete the interfacial reaction since the sedimented particles formed a non-redispersible solid. However, increasing reaction time to three hours, gave the possibility to easily redisperse the dispersion. Two different concentrations of GNP were prepared, separately. A schematic view of the chemical interfacial reaction and structure of the prepared capsules can be seen in Figure 1a.

### 2.4. Photothermal Slurry Preparation

In this study, three photothermal slurries with different concentrations are prepared by dispersing the prepared encapsulated PCMs into water using a magnetic stirrer: Cr60@polyurea, Cr60@polyurea-0.04%GNP, and Cr60@polyurea-0.1%GNP (Figure 1b). Since the results of Cr60@polyurea-0.04%GNP is slightly different from Cr60@polyurea-0.1%GNP, so just the properties of Cr60@polyurea-0.1%GNP are discussed in this paper. These slurries are stable enough for at least 2 h. For longer periods, some sedimentation can be observed which can be easily redispersed by shaking. This shows the applicability of slurries for the stationary state test for up to 2 h, while having enough long-time stability for dynamic state (with pumping) systems.

### 2.5. Characterization Methods

The chemical characterization of the MEPCMs was carried out by a Fourier transform infrared (FTIR, Spectrum 100 spectrometers, PerkinElmer, Shelton, Connecticut, USA) spectrometer for the wave range of 400−4000 cm^−1^. The morphology and microstructure of the capsules were characterized by using scanning electron microscope (SEM, Quanta 600 FEI microscope, Thermo Scientific™ Quanta™, Hillsboro, Oregon, USA). Differential scanning calorimeter (DSC, Mettler Toledo DSC 822^e^, Mettler-Toledo BV, Tiel, Netherlands) was applied to investigate the thermal properties of the samples. For DSC measurements, 4–8 mg of each sample was sealed in an aluminum pan for characterization at a heating rate of 5 °C/min. A thermogravimetric analyzer (TGA/DSC, Mettler Toledo DSC 822^e^) was used to determine the thermal stability and specific heat capacity (Cp) at the constant atmospheric pressure. The TGA analysis was performed with a heating rate of 5 °C/min from 25 to 700 °C in an argon atmosphere with a flow rate of 50 mL/min. Aluminum oxide sample cups of 70 µl were used with approximately 5 mg sample. The optical transmittance spectra (wavelength: 200–2500 nm) of the slurries were measured at room temperature using quartz cuvettes and a 10 mm beam path length by a UV-vis-NIR spectrophotometer (UV-3600, Shimadzu, Long Beach, California, USA). To record the data with the spectrometer the slurries were diluted to 45 ppm.

### 2.6. Photothermal Performance Analysis

**Figure 2:**
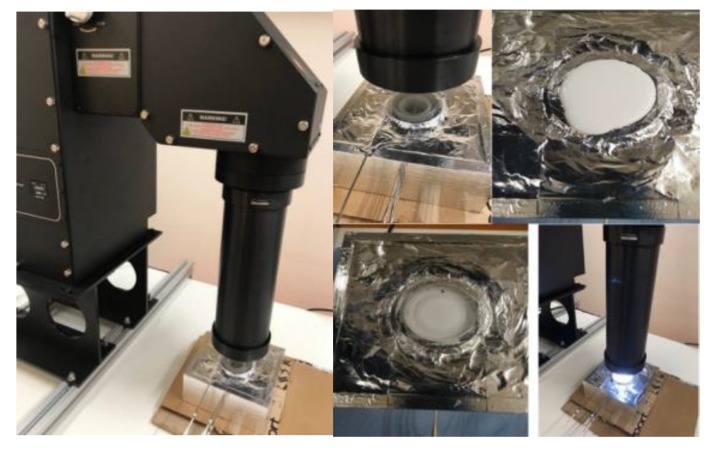
Photo-to-thermal conversion set-up.

A static lab-scale direct absorption solar collector (DASC) set up was built to study the photo-thermal performance of slurries. The experiments were carried using an artificial sunlight simulator as a light source with the capability of up to 7000 ± 0.1 W/m^2^ irradiance that meets class AAA standard and an AM1.5 spectrum (Figure 2). The photo thermal slurries were kept in a 3D printed container, made of polylactic acid (PLA), with an inner diameter of 30 mm and a height of 30 mm. This container was completely insulated to reduce heat loss from the collector walls and the quartz window covering the top of the cylinder. To investigate the temperature distribution of the slurry, six pt100 type thermocouples were adjusted in the middle of cylinder at different heights (y/h = 0, 0.2, 0.4, 0.6, 0.8, and 1). The error of the measured temperatures was evaluated to be ± (0.15 ± 0.002) °C.

## 3. Results and Discussion

### 3.1. Chemical Characterization of Microcapsules

**Figure 3:**
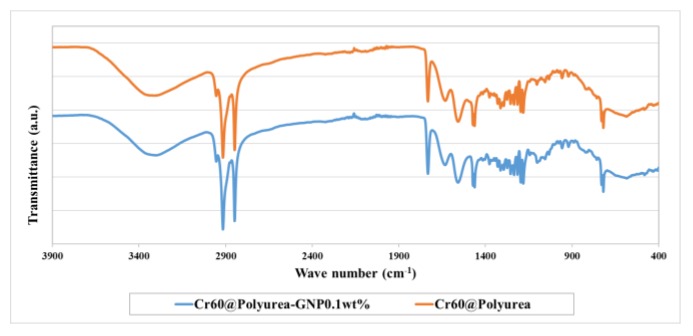
FTIR spectra of Cr60@polyurea-0.1%GNP and Cr60@Polyurea samples.

The FTIR spectra of the prepared samples were studied to check the chemical interaction of the capsules (Figure 3). The peaks observed at 2925 and 2845 cm^−1^ due to the symmetrical stretching vibration of –CH_3_ and –CH_2_ are associated with the PCM. The resulting polyurea polymer used as a microcapsule shell presents the typical vibration bonds of polyurea as depicted in Figure 3. The most important bonds for this specific polymer are the ones resulted from the vibration of N–H and C=O bonds, which are representing the urea linkage. In the FTIR spectra, two bonds can be observed at 1735 and 1633 cm^−1^, related to the stretching of the carbonyl group. The broad bond at 3334 cm^−1^ is related to stretching of NH and to the secondary amines from DETA. The strong bond at 1552cm^−1^ is related to the vibration of the stretching bond N–H from the urea linkage, directly connected to the carbonyl group. The small peak bond presented at 2257 cm^−1^ corresponds to the stretching vibration of the isocyanate group (–NCO), probably due to unpolymerized residues from the diisocyanate (IPDI) that was not fully consumed.

The absorption peaks of the polyurea and Crodatherm 60 can be found in the spectra of encapsulated Cr60@polyurea and Cr60@polyurea-0.1%GNP samples. This means that a combination of wax and polyurea exists in one sample. The absorption peaks from Cr60 are not changed significantly within the prepared samples’ spectra and no new peaks were observed. Therefore, it can be concluded that there is absolutely no chemical interaction between the molecules of Cr60, polyurea, and GNP.

### 3.2. Surface Morphology of Microcapsules 

**Figure 4:**
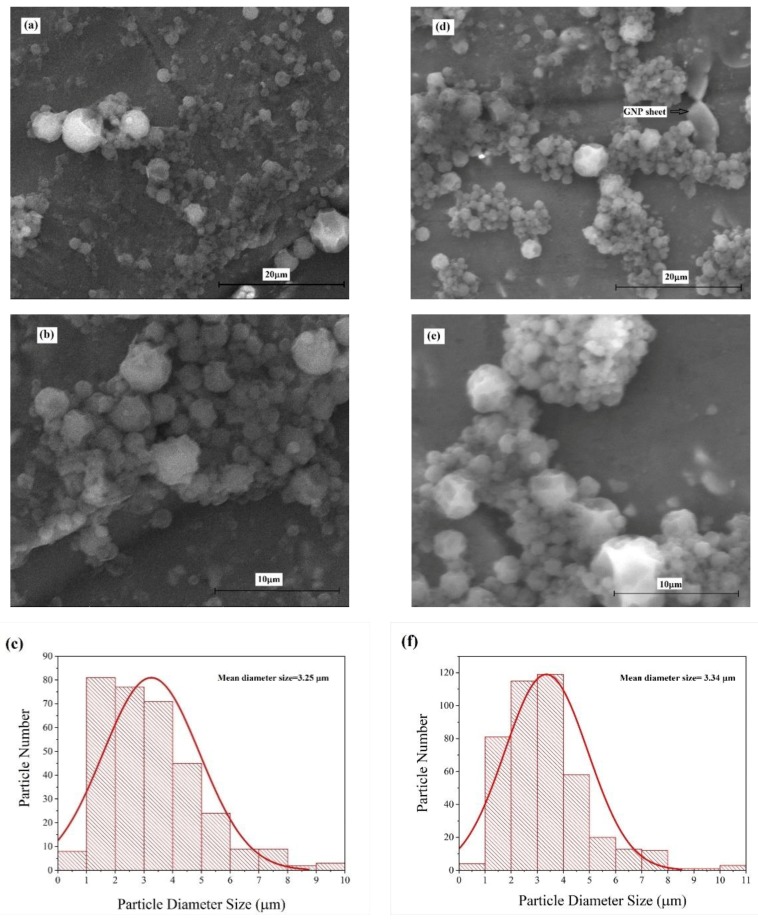
(**a**,**b**) SEM images and; (**c**) particle size distribution of Cr60@Polyurea; (**d**,**e**) SEM images; and (**f**) particle size distribution of Cr60@Polyurea-0.1 wt %GNP.

The SEM photographs of the prepared materials at different magnifications are shown in Figure 4. The SEM photograph of the sample illustrates the spherical structure of material and their smooth surfaces. Particle mean diameter sizes of about 3.25 μm and 3.34 μm were measured for Cr60@polyuea and Cr60@polyurea-0.1%GNP, respectively. The size distribution of samples with GNP are narrower; this indicates more uniformity of particles compared to the ones without GNP. The slight difference of the diameter size and uniformity of capsules between these two samples may have occurred due to the existence of GNP sheets that leads to the fabrication of different lengths of polyurea chains. Some GNP sheets can be observed in the SEM images.

### 3.3. Thermal Characteristics of Microcapsules

**Figure 5:**
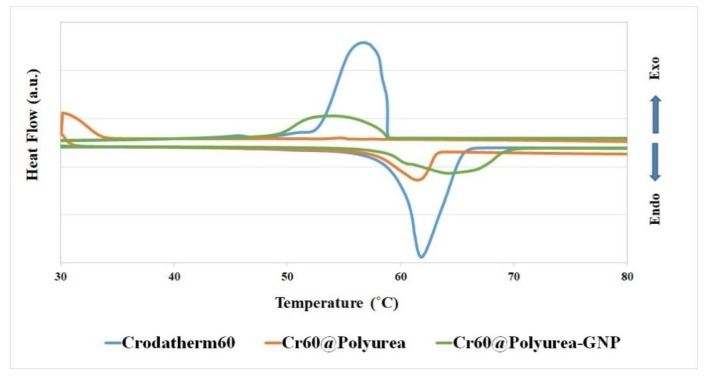
DSC curves of encapsulated Crodatherm60, polyurea, and capsules including polyuria-0.1%GNP.

The thermal characteristics of the MEPCMs directly affect their applications in heat energy storage. The DSC curves of melting and solidifying of Cr60, Cr60@polyurea, and Cr60@polyurea-0.1%GNP are presented in Figure 5. The measurement was done using a Mettler Toledo apparatus at a heating rate of 5 °C/min. The thermal properties, summarized in Table 1, show that the prepared capsules of Cr60@polyurea start melting at 64.6 °C and solidifying at 24.9 °C, while the pure PA start melting at 61.9 °C and solidifying at 56.8 °C. The results indicate that using GNP in the preparation of capsules enhances the encapsulation ratio while decreasing the supercooling from 39.4 °C to 10.4 °C. This acceptable level of supercooling in combination with the achieved latent heat of 95.5 J/g makes the Cr60@polyurea-0.1%GNP capsules suitable for thermal storage applications.

The encapsulation ratio (R) describes the efficient encapsulation of core within the shell. Encapsulation efficiency (E) illustrates the effective performance of the PCMs inside the capsules and the high thermal storage capabilities (φ) of the MEPCMs indicates that nearly all the microcapsules stored latent heat effectively through the phase change (Equation (1)).

(1) $$\{\begin{array}{c} R=\frac{\Delta H_{m,\mathrm{MEPCM}}}{\Delta H_{m.\mathrm{PCM}}}\times 100\%\\ E=\frac{\Delta H_{m,\mathrm{MEPCM}}+\Delta H_{c,\mathrm{MEPCM}}}{\Delta H_{m,\mathrm{PCM}}+\Delta H_{c,\mathrm{PCM}}}\times 100\%\\ \varphi =\frac{E}{R}\times 100\%\; \end{array}$$

As it is shown in Table 1, 98.7% of the encapsulated PCMs are effectively active in the thermal storage process in Cr60@polyurea-0.1%GNP samples.

### 3.4. Thermal Stability of Microcapsules

**Figure 6:**
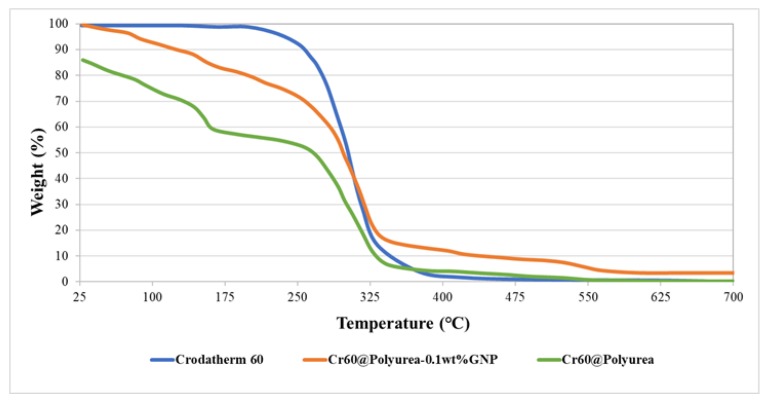
TGA graphs of Cr60, Cr60@polyurea, and Cr60@polyurea-0.1%GNP.

Thermal stability is very important for a reliable performance of encapsulated PCMs in the practical applications. In this study, the thermal stabilities of the pure Crodatherm60, Cr60@polyurea, and Cr60@polyurea-0.1%GNP were evaluated by means of TGA, as shown in Figure 6.

Based on the TGA results (Table 2), all the samples, except for the Crodatherm60, degraded in two steps. Crodatherm 60 loses 100% of its weight at around 304 °C, while Cr60@polyurea and Cr60@polyurea-GNP capsules degrade in two stages. The degradation below 150 °C could have occurred due to the evaporation of water trapped in the system during the microcapsule synthesis.

Thermal decomposition of the shell material may contribute to the weight loss experienced for temperatures higher than 300 °C. It is known that polyurea is characterized by a microphase-separated morphology that consists of hard-segment domains covalently bonded to a soft-segment matrix into a block copolymer architecture. In particular, the hard domains are extensively hydrogen-bonded and serve as reversible physical cross-links, thus providing the material with good mechanical properties. Hence, the second mass loss peak observed in TGA graphs can be due to the thermal degradation of the polyurea shell hard segments, as a result of the relatively low thermal stability of the urea group. However, the degradation temperature of the samples with 0.1 wt % of GNP has been increased 28 °C more compared to Cr60@polyurea capsules. This indicates the effect of using GNP in improving the thermal stability of capsules.

### 3.5. Rheology Behaviour (Viscosity) of the MEPCM Slurry 

**Figure 7:**
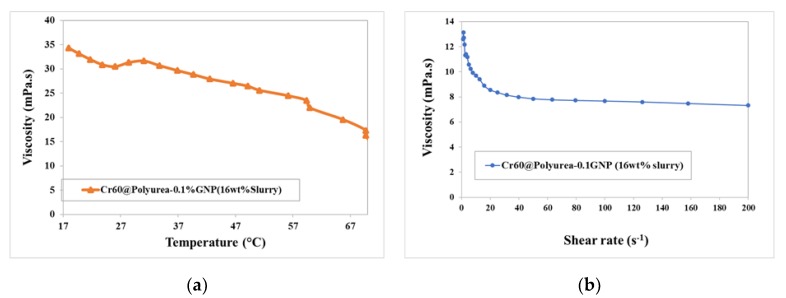
Rheological behavior of 16 wt % Cr60@Polyurea-0.1%GNP slurry (**a**) viscosity versus shear rate at 25 °C; (**b**) viscosity versus temperature at constant shear rate 10 s^−1^.

Viscosity of the slurries is one of the main parameters, which determines the quality of heat transfer fluid. To determine the rheological behavior of the Cr60@Polyurea-0.1%GNP slurry, the viscosity versus shear rate was measured at the constant temperature of 25 °C. Furthermore, a temperature ramp test was conducted at a constant shear stress to investigate the viscosity during the phase transition. The sample was heated up from 17 to 70 °C. The results are shown in Figure 7. As it was expected, by rising the temperature, the viscosity decreases. This can be due to the weakening of the interparticle and intermolecular adhesion forces. By increasing the temperature, the thermal movement of molecules and Brownian motion strengthen, and intramolecular interactions become attenuated [28]. It can be also observed that around the melting temperature (about 64 °C), the characteristic of the viscosity does not change much. This indicates that the phase change process of the entrapped PCMs does not influence the viscosity due to the low seepage of the PCM. Moreover, by increasing the shear rate from 0.1 to 200 s^−1^ (Figure 7b), the viscosity decreases rapidly from 12.5 to about 8 mPa.s., then it becomes constant. This shear thinning behavior can be due to the spatial layout of the microcapsules in the dispersion. It is explained that in a stagnation condition the microcapsules are dispersed randomly in the base fluid. Thus, by shearing the slurry, the microcapsules have unorganized movements not along the bulk flow direction. Afterwards, by increasing the shear rates, the microcapsules start to move between various layers in the main flow direction. Accordingly, the friction between layers decreases, which makes the motion much easier resulting in a lower viscosity.

Even though the viscosity of the sample is about 8 to 14 times higher than that of water, the prepared slurry has a low viscosity which meets the transportability requirements in pump systems for heat transfer applications.

### 3.6. Optical Characterization of the MEPCM Slurry (UV-Vis-NIR)

**Figure 8:**
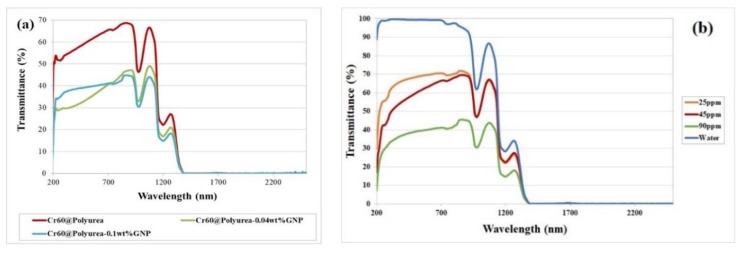
Transmittance spectra of (**a**) Cr60@polyurea, Cr60@polyurea-0.1 wt %GNP, and Cr60@polyurea-0.04 wt %GNP slurries at 45 ppm concentration; (**b**) slurries of water and Cr60@polyurea-0.1 wt %GNP with different concentrations.

The optical transmittance spectra (wavelength: 200–2500 nm) of the slurries have been measured at a room temperature using quartz cuvettes and a 10 mm beam path length. The results are shown in Figure 8. To record the data with the spectrometer, the slurries were diluted to 45 ppm. Water shows the well-known behavior with an average transmittance of about 96% in the wavelength range of 200–900 nm, while typical absorption peaks exist at 900–1000 nm and at 1200 nm. In the wavelength range of 200 to 900 nm, the absorbance effect of carbon materials is illustrated in Figure 8 as well. The GNP nanofluids at different concentrations maintain an analogous behavior compared to the base fluid, except for the occurrence of an absorption valley around a wavelength of 270 nm due to the strong absorption of carbon material. By increasing the concentration of GNP in GNP nanofluids, the radiation absorption enhanced as indicated in Figure 8b. The optical behavior of the materials indicated that the carbon materials in the slurries enhance the solar radiation absorption of the slurries.

### 3.7. Photothermal Measurement 

**Figure 9:**
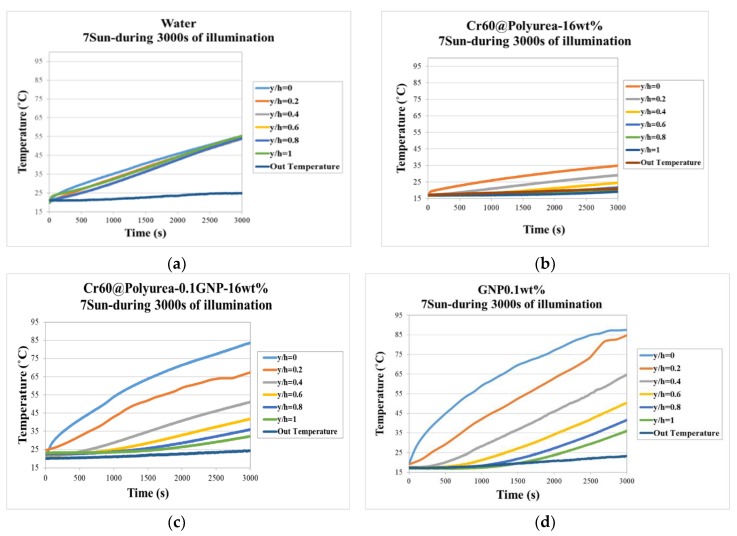
Photo-to-thermal conversion spectra of (**a**) water; (**b**) 0.1%GNP; (**c**) Cr60@Polyurea; and (**d**) Cr60@polyurea-0.1%GNP samples (h = the total height of the collector, y = the height distance from the illumination surface to the location of the adjusted thermocouple).

The working fluid in the DASC system absorbs the sunlight and converts its energy into heat. This will increase the fluid temperature, depending on its optical absorption capability, as well as the thermal conductivity and heat capacity. The photo-thermal conversion performance of water and the prepared slurries were tested in this section using illumination intensity of 7000 ± 0.1 W/m^2^ as shown in Figure 9.

Figure 9c shows the light to heat conversion graphs of the Cr60@polyurea-0.1%GNP samples. It takes 3000 s to increase the temperature of the 16 wt % Cr60@polyurea-0.1%GNP slurry from room temperature (around 20 °C) to 85 °C. While under the same exposure time, the temperature of the 16 wt % Cr60@polyurea and water raise to 35 °C and 55 °C, respectively (Figure 9a,b). That can be explained by the poor solar light absorption of water and the white slurry in comparison with that of the gray one. Due to the presence of GNP, the time required to reach 85 °C has been greatly reduced. By dispersing the same weight ratio of GNP into the water to make nanofluid, the photo-thermal behavior of the receiver could be almost the same.

**Figure 10:**
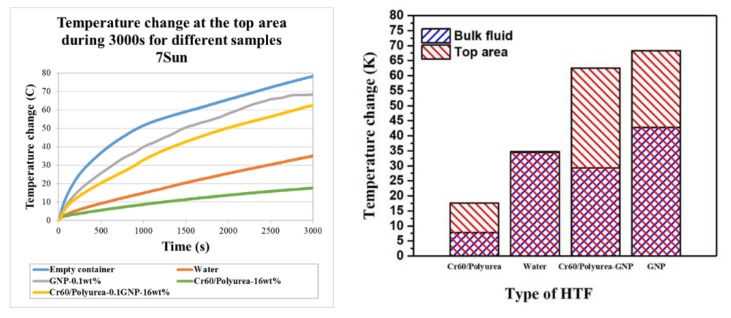
Temperature change difference of Cr60@polyurea-0.1%GP samples.

Considering the temperature profile of different thermocouples in Figure 9 and Figure 10, the temperature distribution of the prepared slurries is not as uniformed as water. However, the top surface temperature of the slurry is much higher than the same condition as water. During the same illumination time, the rising temperature of the Cr60@polyurea samples were lower compared to that of water. This occurred due to the larger heat capacity of water compared to that of the samples without GNP. The uniformity of the temperature distribution of the slurry will be enhanced by the optimum concentration in a pumping system. Outcomes prove that the Cr60@polyurea-GNP slurry has a full absorbance property through the visible wavelength, which is substantial for collecting sunlight irradiation effectively. This fact can be explained due to the function of carbon black as an effective photon captor and molecular heater. The decrease in the slope of the temperature-time graphs after reaching above melting temperature of PCM can be related to the thermal storage performance part of the slurries.

### 3.8. Receiver Efficiency

The efficiency of a collector can be determined as the ratio of the amount of energy transferred from the collector to the heat transfer fluid to the incident radiant energy on the collector. Therefore, to further study the photo-thermal performance of the prepared slurries in a simulated DASC system, the efficiency of the receiver can then be calculated as:

(2) $$\eta =\frac{m\int^{\text{​}}C\left(T\right)\mathrm{dT}}{\mathrm{GAt}}=\frac{m_{\mathrm{total}}\cdot C_{\mathrm{slurry}}\cdot \Delta T+(m_{\mathrm{melted}\;\mathrm{capsules}}\cdot \Delta H_{f,\mathrm{capsules}})}{G\cdot A\cdot t}\;\Delta T=T\left(t\right)-T\left(\mathrm{initial}\right)$$

where T (initial) (°C) is the initial temperature of the fluid, G (W/m^2^) is the heat flux of the incident light from the solar simulator, which was measured to be about 7000 W/m^2^ in this research, t (s) is the irradiation exposure time, and A (m^2^) is the illumination surface area [13].

**Figure 11:**
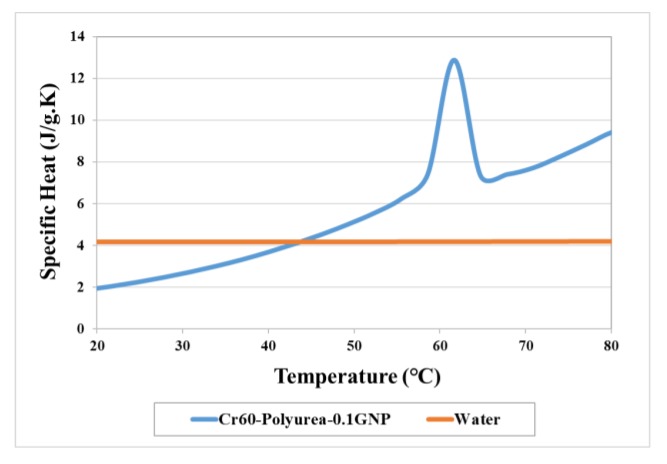
Specific heat of Cr60@polyurea-0.1%GNP slurry (16 wt %) and water [30].

The specific heat capacity of the prepared Cr60@polyurea-0.1%GNP slurry was measured by using the DSC instrument. Figure 11 demonstrates the specific heat capacity of this sample in comparison with that of water.

The specific heat capacity of water moderately decreases from 4.21 to 4.19 J/g.K as temperature rises from 20 to 80 °C based on the previous studies (Figure 11) [29]. When dispersing 16 wt % Cr60@polyurea-0.1%GNP particles into the base fluid, the specific heat was largely improved in the melting process (from 55 to 75 °C). The specific heat of the Cr60@polyurea-0.1%GNP increases from 1.8 up to 13 J/g.K during the melting process. It illustrates that the thermal storage capacity of the prepared heat transfer fluid will be improved by means of introducing MEPCMs. This is because of the high energy storage capacity of MEPCSs during the melting and solidifying process, which is consistent with the results presented in Table 1.

The recorded temperature-time behavior of the samples indicated that after almost 4000 s the temperature of the first two thermocouples goes above the melting temperature of the slurry. This means that 40% of the slurry is acting as the thermal storage element (see Figure 9c). Based on the temperature-time graphs and the measured specific heat capacity, the receiver efficiency of the slurry in the simulated DASC can be calculated as:

(3) $$\eta =\frac{\frac{m}{5}\times (\int_{T_{\mathrm{initial}}}^{\frac{T_{\frac{y}{h}=0}+T_{\frac{y}{h}=0.2}}{2}}C_{p}\cdot \mathrm{dT}+\int_{T_{\mathrm{initial}}}^{\frac{T_{\frac{y}{h}=0.2}+T_{\frac{y}{h}=0.4}}{2}}C_{p}\cdot \mathrm{dT}+\int_{T_{\mathrm{initial}}}^{\frac{T_{\frac{y}{h}=0.4}+T_{\frac{y}{h}=0.6}}{2}}C_{p}\cdot \mathrm{dT}+\int_{T_{\mathrm{initial}}}^{\frac{T_{\frac{y}{h}=0.6}+T_{\frac{y}{h}=0.8}}{2}}C_{p}\cdot \mathrm{dT}+\int_{T_{\mathrm{initial}}}^{\frac{T_{\frac{y}{h}=0.8}+T_{\frac{y}{h}=1}}{2}}C_{p}\cdot \mathrm{dT})}{G\cdot A\cdot t}$$

Looking at a receiver with 3 cm height after 3000 s, no changes in the efficiency of utilized slurry was observed compared to the water, which was about 21% for each case. While considering a receiver of 1.2 cm long in the similar condition, efficiency increased to 30% by introducing slurry in the system. The efficiency was however equal to 9% when the receiver was filled by pure water. This can be explained by the higher temperature achieved at the surface of the receiver when using slurry due to the presence of GNP particles, which can be seen in Figure 9b,c. The energy density in case of introducing slurry will be 4 times more than that of the water. Regardless of the changes in the receiver efficiency, by introducing PCM into the system, the bulk temperature of the receiver and therefore, the heat losses, were reduced which is a very important factor to be considered in designing the DASC systems.

## 4. Conclusions

In this research, Crodatherm^TM^ 60 as a thermal energy storage material was successfully encapsulated in polyurea by utilizing an interfacial reaction. Graphite nanoplatelets were incorporated to enhance the thermal and photothermal performance of the MEPCMs in an aqueous dispersion. The microstructure and morphology of the prepared capsules were characterized by SEM and FTIR spectrum. The results showed the successful fabrication of core-shell spherical type of MEPCMS with negligible leakage during the melting process. No chemical interaction between the core and the supporting materials was observed. The thermal characteristics of the MEPCMs, analyzed by DSC and TGA, show that the Crodatherm60@polyurea-0.1%GNP possess the latent heat of 95.5 J/g at the phase transition temperature of about 64 °C. Furthermore, a stable dispersion of 16 wt % of the MEPCMs into water illustrated improved photothermal conversion performance when temperature increased from 17 to 85 °C. Analyzing the rheological properties of the prepared slurry showed that the prepared materials meet the transportability requirements in pump systems for heat transfer applications. The direct solar radiation absorption behavior of slurries was confirmed. The optical behavior of the materials indicated that the carbon material enhances the solar radiation absorption of the slurry. The photothermal behavior of the slurry was investigated by using a simulated static state direct absorption solar collector. Outcomes indicated that for a receiver of 1.2 cm height, the efficiency increased to 30% by introducing slurry in the system, while the efficiency was equal to 9% when the receiver was filled by pure water. Together with the changes in the receiver efficiency, by introducing PCM into the system, the bulk temperature of the receiver and therefore the heat losses were reduced. The thermal storage capacity, good thermal stability, and enhanced photothermal performance of the prepared slurries make them a good candidate for practical applications.

## Figures and Tables

**Table 1:** Thermal properties of pure and encapsulated Cr60.

Sample Code	T_m,peak_ (°C)	ΔH_m_ (J/g)	T_c,peak_ (°C)	ΔH_c_ (J/g)	Encapsulation Ratio (%)	Encapsulation Efficiency (%)	Thermal Storage Capacity (%)
Cr60	61.9	218.9	56.8	206.0	-	-	-
Cr60@Polyurea	64.6	61.6	25.2	24.9	28.1	20.4	72.3
Cr60@Polyurea-0.1%GNP	64.3	95.5	53.9	87.5	43.6	43.1	98.7

**Table 2:** Thermogravimetric analysis data of the prepared capsules.

Sample Code	∆m_tot_ (%)	∆m_Step1_ (%)	∆m_Step2_ (%)	T_p1_ (°C)	T_0_ (°C)	T_p2_ (°C)
Cr60@polyurea-0.1 wt %GNP	−96	−21	−75	155	280	315
Cr60@polyurea	−92	−28	−59	153	252	312
Crodatherm60	−100	-	−95	-	-	304
